# Efficacy and safety of aflibercept biosimilars compared to aflibercept in the treatment of neovascular age-related macular degeneration: a systematic review and meta-analysis

**DOI:** 10.3389/fphar.2026.1719493

**Published:** 2026-02-17

**Authors:** Yating Zhou, Zongyue Zhan, Zixun Wang, Chen Liu

**Affiliations:** 1 Department of Ophthalmology, Kunshan Hospital of Traditional Chinese Medicine, Suzhou, Jiangsu, China; 2 Department of Ophthalmology, Shanghai University Affiliated Heping Eye Hospital, Shanghai, China; 3 Tianjin Key Laboratory of Retinal Functions and Diseases, Tianjin Branch of National Clinical Research Center for Ocular Disease,Eye Institute and School of Optometry, Tianjin Medical University Eye Hospital, Tianjin, China; 4 Department of Ophthalmology, Affiliated Xinhua Hospital of Dalian University, Dalian, Liaoning, China

**Keywords:** aflibercept, anti-vascular endothelial growth factor therapy, best-corrected visual acuity, biosimilars, meta-analysis, neovascular age-related macular degeneration, randomized controlled trials

## Abstract

**Background:**

This study aimed to evaluate the aflibercept biosimilars compared to the reference product aflibercept (Eylea®) in terms of efficacy, safety, and immunogenicity in patients with neovascular age-related macular degeneration (nAMD).

**Methods:**

We searched PubMed, Web of Science, Cochrane Library, and Embase from inception to 13 August 2025. We included studies reporting changes in best-corrected visual acuity (BCVA), changes in central subfield thickness (CST), changes in the leakage lesion of choroidal neovascularization (CNV), and adverse events from baseline to endpoint. All statistical analyses were performed using Stata 18.0 software and assessed the certainty of evidence for each outcome using the GRADE approach.

**Results:**

A total of seven studies involving 2,829 participants were included. There were no statistically significant differences in visual and anatomical outcomes between the aflibercept biosimilars (SB15, P041, SDZ-AFL, AVT06, SCD411, ABP 938, and QL1207) and the reference aflibercept. No significant differences were detected between aflibercept biosimilars and the reference aflibercept with respect to serious ocular and non-ocular adverse events [relative risk (RR) = 1.71, 95% confidence interval (CI): 0.70, 4.19; *I* ^*2*^ = 0.0%, *P* = 0.913; RR = 1.08, 95% CI: 0.82, 1.42; *I* ^*2*^ = 0.0%, *P* = 0.936, respectively). Although a slightly higher rate of treatment-emergent adverse events (TEAEs) was noted in the biosimilar group (RR = 1.07, 95% CI: 1.00–1.15; I^2^ = 26.1%), the difference was not statistically significant (*P* = 0.248).

**Conclusion:**

Based on the seven included randomized controlled clinical trials, aflibercept biosimilars demonstrated comparable safety and efficacy to the reference aflibercept. Future research requires more rigorous studies.

## 1 Introduction

Neovascular age-related macular degeneration (nAMD) affects millions of individuals worldwide, representing the most common cause of severe visual impairment in patients over 75 years of age and one of the leading causes of blindness globally (Klein et al., 2007). It is projected that the global prevalence of nAMD will rise to approximately 288 million by 2040 (GBD 2021 Global AMD Collaborators, 2025). Vision loss in nAMD is primarily caused by choroidal neovascularization (CNV), which is characterized by the abnormal growth of blood vessels that leak fluid and blood into the retina, leading to edema, scarring, and metamorphopsia (Deng et al., 2022; Flores et al., 2021; Ferris et al., 2013). This pathological angiogenesis is mediated by vascular endothelial growth factor (VEGF), which plays a critical role in the development of CNV in nAMD. Dysregulated expression of specific VEGF isoforms, induced by hypoxia, contributes to leakage and hemorrhage in nAMD (Homayouni, 2009).

Current treatment modalities include intravitreal anti-VEGF therapy, laser treatment, and surgical intervention. Among these, intravitreal injection of anti-VEGF agents—such as ranibizumab, aflibercept, and faricimab—constitutes the first-line clinical standard for nAMD. These therapeutics block the signaling molecules that stimulate pathological blood vessel growth and reduce vascular leakage, thereby yielding favorable visual outcomes (Thomas et al., 2021). Aflibercept is a recombinant fusion protein that binds to VEGF receptors and placental growth factor, thereby inhibiting angiogenesis (Dorrell et al., 2007). The injections can be administered efficiently in an outpatient setting with minimal procedure time, requiring almost no recovery period for patients and presenting few risks or side effects (Lau et al., 2018).

Although anti-VEGF therapies have revolutionized the management of nAMD, their high cost imposes a substantial economic burden on both patients and healthcare systems. With a growing aging population, these expenses are expected to increase exponentially. Thus, providing effective and affordable therapeutic alternatives is imperative. Biosimilars are not simple generic drugs but are biological products that undergo a rigorous, stepwise, and comprehensive scientific evaluation to demonstrate high similarity to the reference licensed biologic product in terms of physicochemical properties and biological activity, with no clinically meaningful differences in safety, purity, and potency (US Food and Drug Administration, 2015). With the expiry of the reference product patents for aflibercept in 2023 in the United States and China and expected expiry in Europe in 2025, opportunities are emerging for the entry of biosimilars into these markets. Following the loss of exclusivity, multiple aflibercept biosimilars are under active development, with market approvals anticipated in the coming years. Biosimilars present a significant opportunity to reduce healthcare costs while ensuring broader patient access to treatments that are equally safe and efficacious as the reference product.

Aflibercept (Eylea®; Regeneron), a recombinant fusion protein composed of the extracellular domains of VEGF receptors 1 and 2 fused to the Fc portion of IgG1, binds both VEGF-A and placental growth factor (Holash et al., 2002). The VIEW 1 and VIEW 2 studies demonstrated that aflibercept is non-inferior to ranibizumab for the treatment of nAMD and support its widespread use across multiple regions, including the United States, Europe, and China (Heier et al., 2012; Marques et al., 2022). However, the high cost and frequent dosing schedule of aflibercept impose a substantial economic burden on patients and healthcare systems. Biosimilars offer a cost-effective alternative to originator biologics. These agents are highly similar in structure, function, and clinical efficacy, with no meaningful differences in terms of safety or immunogenicity. With aflibercept patents expiring in the US and China in 2023 and in Europe by 2025, several biosimilar candidates have been developed and approved globally, including SB15 (Opuviz), ABP 938 (Pavblu), SDZ-AFL (SOK583A1), P041 (Tyalia), AVT06, Yesafili, Ahzantive (FYB203), Enzeevu, and Afqlir (Aljuhani et al., 2025). The increased availability of these biosimilars offers an opportunity to expand patient access and reduce the financial burden associated with long-term nAMD management (Dutta et al., 2020; Carl et al., 2022; Mulcahy et al., 2022). Despite promising results from individual clinical trials, a comprehensive evaluation of the clinical performance of aflibercept biosimilars is warranted. The purpose of this meta-analysis is to investigate and evaluate the benefits and risks of aflibercept biosimilars versus the reference product in patients with nAMD.

## 2 Methods

This meta-analysis was conducted in accordance with the Preferred Reporting Items for Systematic Reviews and Meta-Analyses (PRISMA) guidelines (Page et al., 2021) and was registered under the ID number CRD420251139994. Ethical approval was not required as this is a systematic review.

### 2.1 Search strategy

The following electronic databases were comprehensively searched: PubMed, Web of Science, Cochrane Library, and Embase from 1 January 2005 to 13 August 2025. The search strategy incorporated both MeSH terms and keywords, including “neovascular age-related macular degeneration,” “biosimilar aflibercept,” and “randomized controlled trial.” To ensure literature saturation, ongoing clinical trials were identified through the WHO International Clinical Trials Registry Platform (WHOICTRP) and ClinicalTrials.gov. Preprint servers such as medRxiv and Research Square were also screened to identify unpublished data. More details about the search strategy of PubMed are shown in Table 1; analogous strategies were applied to the remaining databases.

**TABLE 1:** Search strategy of PubMed.

Search number	Query	Results
#1	“Macular Degeneration” [Mesh]	33,133
#2	(“Macular Degeneration” [Mesh]) OR (((((((((((((((((((degeneration, Macular [Title/Abstract]) OR (macular Degenerations [Title/Abstract])) OR (Maculopathy [Title/Abstract])) OR (Maculopathies [Title/Abstract])) OR (macular Dystrophy [Title/Abstract])) OR (dystrophy, Macular [Title/Abstract])) OR (macular Dystrophies [Title/Abstract])) OR (age-related macular Degeneration [Title/Abstract])) OR (age related macular Degeneration [Title/Abstract])) OR (age-related macular Degenerations [Title/Abstract])) OR (macular degeneration, Age-Related [Title/Abstract])) OR (macular degeneration, age Related [Title/Abstract])) OR (maculopathies, Age-Related [Title/Abstract])) OR (maculopathy, Age-Related [Title/Abstract])) OR (maculopathy, age Related [Title/Abstract])) OR (age-related Maculopathies [Title/Abstract])) OR (age related Maculopathies [Title/Abstract])) OR (age-related Maculopathy [Title/Abstract])) OR (age related Maculopathy [Title/Abstract]))	48,030
#3	((((((biosimilar*[Title/Abstract]) OR (biobetter*[Title/Abstract])) OR (biomimic*[Title/Abstract])) OR (biogeneric*[Title/Abstract])) OR (biologic*[Title/Abstract])) OR (similar*[Title/Abstract])) OR (biotherapeutic*[Title/Abstract])	3,865,135
#4	(((((((((((((((((((((((((((((((((((((((((((((((((((((((Aflibercept*[Title/Abstract]) OR (abp 938 [Title/Abstract]) OR (abp938 [Title/Abstract])) OR (alt l9 [Title/Abstract])) OR (altl9 [Title/Abstract])) OR (ave 0005 [Title/Abstract])) OR (ave0005 [Title/Abstract])) OR (ave 005 [Title/Abstract])) OR (ave005 [Title/Abstract])) OR (avt 06 [Title/Abstract])) OR (avt06 [Title/Abstract])) OR (bay 86 5321 [Title/Abstract])) OR (bay 865321 [Title/Abstract])) OR (bay865321 [Title/Abstract])) OR (chs 2020 [Title/Abstract])) OR (chs2020 [Title/Abstract])) OR (CT-P42 [Title/Abstract])) OR (CTP42 [Title/Abstract])) OR (eylea [Title/Abstract])) OR (eylia [Title/Abstract])) OR (fyb 203 [Title/Abstract])) OR (fyb203 [Title/Abstract])) OR (gbs 012 [Title/Abstract])) OR (gbs012 [Title/Abstract])) OR (ly 01012 [Title/Abstract])) OR (ly01012 [Title/Abstract])) OR (ly 09004 [Title/Abstract])) OR (ly09004 [Title/Abstract])) OR (myl 1701p [Title/Abstract])) OR (myl1701p [Title/Abstract])) OR (ot 702 [Title/Abstract])) OR (ot702 [Title/Abstract])) OR (pbp 1,602 [Title/Abstract])) OR (pbp1602 [Title/Abstract])) OR (pmc 902 [Title/Abstract])) OR (pmc902 [Title/Abstract])) OR (ql 1,207 [Title/Abstract])) OR (ql1207 [Title/Abstract])) OR (regn 3 [Title/Abstract])) OR (regn3 [Title/Abstract])) OR (sb 15 [Title/Abstract])) OR (sb15 [Title/Abstract])) OR (scb 420 [Title/Abstract])) OR (scb420 [Title/Abstract])) OR (scd 411 [Title/Abstract])) OR (scd411 [Title/Abstract])) OR (sok 583a1 [Title/Abstract])) OR (sok583a1 [Title/Abstract])) OR (SOK583A19 [Title/Abstract])) OR (SOK 583 A19 [Title/Abstract])) OR (syn 112 [Title/Abstract])) OR (syn112 [Title/Abstract])) OR (vascular endothelial growth factor trap [Title/Abstract])) OR (vasculotropin trap [Title/Abstract])) OR (VEGF Trap [Title/Abstract])) OR (wetlia [Title/Abstract])) OR (zaltrap [Title/Abstract])	4,355
#5	#3 OR #4	3,869,012
#6	((((((Randomized controlled trial [Publication type]) OR (randomized [Title/Abstract])) OR (controlled clinical trial [Publication type]))) OR (placebo [Title/Abstract])) OR (randomised [Title/Abstract])) OR (randomized [Title/Abstract])	1,284,459
#7	#2 AND #5 AND #6	748

### 2.2 Inclusion and exclusion criteria

Studies were included if they met the following criteria: (1) randomized controlled trials (RCTs) comparing intravitreal aflibercept biosimilars to the reference aflibercept product; (2) enrollment of patients with untreated subfoveal CNV secondary to nAMD; (3) participants aged 50 years or older; and (4) reported data on any of the following outcomes: best-corrected visual acuity (BCVA), central subfield thickness (CST), changes in the leakage lesion of CNV, adverse events (serious ocular and serious non-ocular AEs during the study period), treatment-emergent adverse events (TEAEs) during the study period, and cumulative incidence of anti-drug antibodies (ADA). The exclusion criteria comprised (1) non-randomized studies, reviews, case reports, or animal studies; (2) studies evaluating interventions other than the specified biosimilar and reference aflibercept comparisons; (3) cases of CNV attributable to other underlying conditions; and (4) (Flores et al., 2021) articles published in languages other than English.

### 2.3 Study selection

All identified studies were managed using EndNote 21. After removing duplicates, two independent reviewers (Zy. Z and Zx. W) performed initial screening of titles and abstracts to exclude records that did not meet the inclusion criteria. The full texts of the remaining articles were subsequently retrieved and evaluated for eligibility. Any discrepancies between the reviewers were resolved through discussion or by consultation with a third reviewer (C. L).

### 2.4 Data collection and extraction

Each type of dataset was independently extracted by two authors (C. L and Yt. Z), with conflicts resolved by a third author (Zx. W). The extracted data included (1) first author, year of publication, study design, the number of patients, name of the biosimilar, and mean age; (2) primary outcome: change in BCVA from baseline to endpoint, adverse events (serious ocular and serious non-ocular AEs during the study period), and TEAEs; (3) secondary outcome: change in CST and CNV from baseline to endpoint and the cumulative incidence of ADA. We did not estimate values based on charts or graphs. If no direct data were available in the published study, the research findings were extracted from the original data. In addition, to obtain missing outcome data, we attempted to contact the study authors via email.

### 2.5 Risk-of-bias assessments

The risk of bias and certainty of evidence were assessed by two blinded, independent reviewers (Zy. Z and Zx. W). Disagreement between the two reviewers was resolved through a third independent author (Yt. Z). The RoB 2.0 tool (Stewart et al., 2015) evaluates five key domains of potential bias: the randomization process, deviations from intended interventions, missing outcome data, measurement of the outcome, and selection of the reported result. In addition, the Grading of Recommendations, Assessment, Development, and Evaluations (GRADE) framework (Atkins et al., 2004) rating was established based on the study design: RCTs started as high certainty rating, whereas observational studies began with a low certainty rating. This preliminary rating was then systematically downgraded based on the presence of limitations in any of the following areas: risk of bias, inconsistency, indirectness, imprecision, or publication bias. The final certainty of evidence was categorized into one of four levels: high, moderate, low, or very low. The entire process was conducted using the GRADEpro Guideline Development Tool software, ensuring transparency and reproducibility in the evaluation of evidence quality.

### 2.6 Statistical analysis

Stata 18.0 software was used for meta-analysis. For dichotomous variables, analysis was performed using relative risk (RR) and 95% confidence interval (CI). For continuous variables, standard mean difference (SMD) and 95% CI were chosen to be analyzed. *I* ^*2*^ statistics assess heterogeneity (Higgins et al., 2003). *I* ^*2*^ > 75% is recognized as significant heterogeneity, 50% < *I* ^*2*^ ≤ 75% as moderate heterogeneity, 25% < *I* ^*2*^ ≤ 50% as low heterogeneity, and *I* ^*2*^ ≤ 25% as homogeneity. We used the fixed-effects model if heterogeneity was low or homogeneous. Otherwise, we chose the random-effects model. Sensitivity analysis was performed to detect the source of heterogeneity. Moreover, we used funnel plots and Egger’s regression test to assess the publication bias.

## 3 Results

### 3.1 Study selection

**FIGURE 1:**
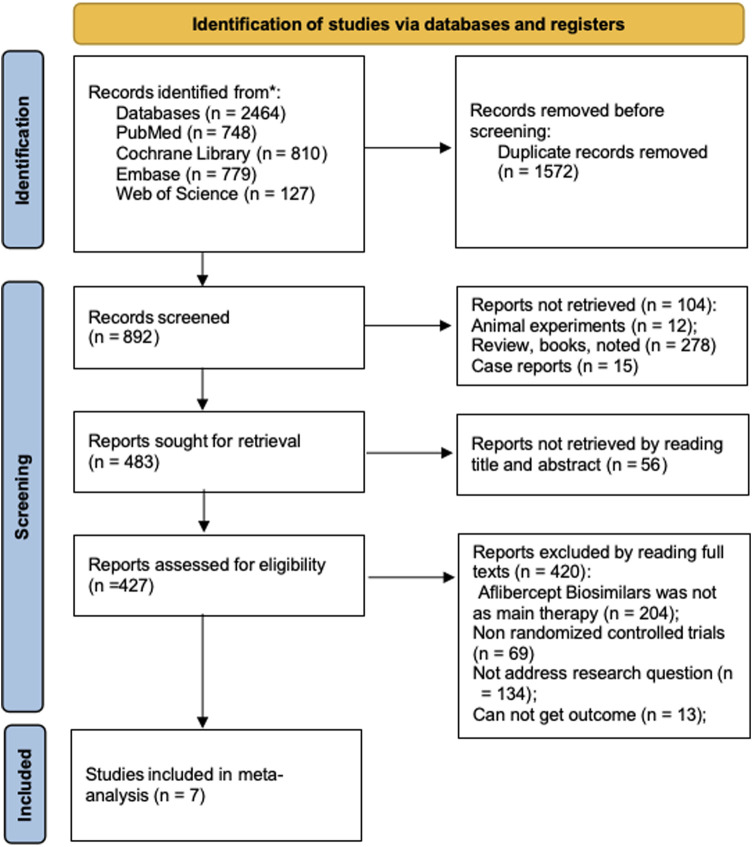
Flow diagram of the selection process.

According to the search strategy, a total of 2,464 potential studies were identified from the four databases. After removing 1,572 duplicate records, the remaining studies were assessed for inclusion by reviewing their titles and abstracts. Following a full-text review, seven RCTs were included based on the eligibility criteria (Sadda et al., 2023; Karkhaneh et al., 2024; Bordon et al., 2024; Agostini et al., 2025; Kang et al., 2024; Friedman et al., 2025; Li et al., 2024). All included studies were phase 3, multicenter, double-masked, active comparator-controlled trials. The biosimilars evaluated were SB15, P041, SDZ-AFL, AVT06, SCD411, ABP 938, and QL1207. All included trials adopted a consistent dosing regimen in which patients received intravitreal injections at 4-week intervals for the first three doses, followed by injections every 8 weeks thereafter throughout the study period. The flow diagram of the study selection process is shown in Figure 1. A total of 2,829 patients with nAMD were enrolled. Among them, 1,348 received the reference aflibercept and 1,481 received the aflibercept biosimilars. Their baseline characteristics are presented in Table 2.

**TABLE 2:** Key demographics and ocular characteristics of the studies included (n = 7).

First author, published year	Study design	Drug: intervention/control		Patients: intervention/control	Age: intervention/control [mean (SD)]	Male, no.: intervention/control (%)	BCVA, ETDRS letter score: intervention/control [mean (SD)]	CST, μm: intervention/control [mean (SD)]	Area of CNV, mm^2^: intervention/control [mean (SD)]	Main outcome measures
Sadda et al. (2023)	56-week, active comparator-controlled, double-masked, phase 3 randomized clinical study performed at 56 sites	SB15	Reference aflibercept	224/225	73.7 (8.1)/74.3 (8.1)	106 (47.3)/93 (41.3)	59.5 (10.6)/58.9 (11.2)	353.3 (95.61)/382.3 (121.96)	6.1 (4.3)/6.3 (4.8)	Mean change in BCVA, CST, CNV, and ADA from baseline to week 32; TEAEs and adverse events (ocular and non-ocular)
Karkhaneh et al. (2024)	52-week, active comparator-controlled, double-masked, phase 3 randomized clinical study performed at 12 centers	P041	Reference aflibercept	84/84	68.64 (6.88)/68.26 (6.24)	58 (69.05)/47 (55.95%)	49.58 (13.82)/54.11 (13.60)	483.57 (207.22)/447.40 (121.68)	NA	Mean change in BCVA, CST, CNV, and ADA from baseline to week 52; TEAEs and adverse events (ocular and non-ocular)
Bordon et al. (2024)	52-week, active comparator-controlled, double-masked, phase 3 randomized clinical study performed at 103 sites	SDZ-AFL	Reference aflibercept	243/240	75.8 (7.82)/75.7 (7.72)	106 (43.6)/104 (43.3)	59.7 (10.05)/59.4 (10.37)	493.8 (169.25)/471.8 (163.85)	5.7590 (5.03035)/5.4383 (4.62344)	Mean change in BCVA, CST, CNV, and ADA from baseline to week 52; TEAEs and adverse events (ocular and non-ocular)
Agostini et al. (2025)	52-week, active comparator-controlled, double-masked, phase 3 randomized clinical study performed at 117 sites	AVT06	Reference aflibercept	205/205	73.7 (9.11)/74.3 (8.04)	191 (46.6)/219 (53.4)	55.8 (11.72)/54.2 (12.38)	430.9 (117.46)/436.2 (128.12)	6.424 (4.9527)/6.469 (4.6831)	Mean change in BCVA, CST, CNV, and ADA from baseline to week 52; TEAEs
Kang et al. (2024)	52-week, active comparator-controlled, double-masked, phase 3 randomized clinical study performed at 117 sites	SCD411	Reference aflibercept	287/286	73.5 (8.0)/73.6 (8.6)	138 (48.1)/139 (48.6)	58.6 (10.8)/59.9 (10.6)	500.5 (184.0)/479.7 (160.1)	4.69 (4.29)/4.44 (4.17)	Mean change in BCVA, CST, CNV, and ADA from baseline to week 52; adverse events (ocular and non-ocular)
Friedman et al. (2025)	52-week, active comparator-controlled, double-masked, phase 3 randomized clinical study performed at 102 sites	ABP 938	Reference aflibercept	288/288	76.0 (7.9)/76.0 (8.0)	137 (47.6)/117 (40.6)	58.9 (10.68)/57.6 (11.74)	438.4 (129.05)/448.8 (128.12)	8.508 (5.6621)/9.343 (5.2270)	Mean change in BCVA, CST, and CNV from baseline to week 52
Li et al. (2024)	52-week, active comparator-controlled, double-masked, phase 3 randomized clinical study performed at 35 sites in China	QL1207	Reference aflibercept	185/181	67.4 (8.9)/67.1 (8.0)	122 (65.9)/126 (69.6)	56.1 (11.7)/56.3 (11.8)	428.2 (178.1)/463.5 (193.3)	3.99 (4.14)/3.99 (4.13)	Mean change in CST, CNV, and ADA from baseline to week 52; TEAEs and adverse events (ocular and non-ocular)

Abbreviations: BCVA, best-corrected visual acuity; CNV, choroidal neovascularization; CST, central subfield thickness; ETDRS, Early Treatment Diabetic Retinopathy Study; TEAEs, treatment-emergent adverse events; ADA, anti-drug antibodies.

### 3.2 Quality assessment and risk of bias

**FIGURE 2:**
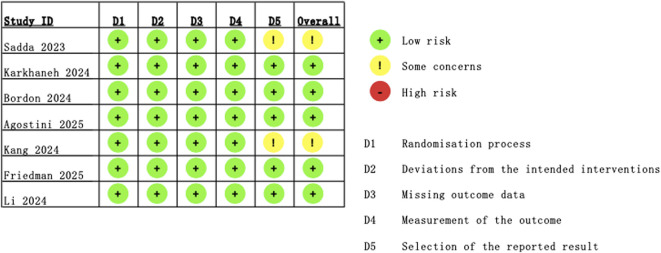
Traffic light plot of the risk-of-bias assessment of RCTs using RoB 2.0. RoB 2.0, Risk of Bias 2.0; RCTs, randomized controlled trials.

**FIGURE 3:**
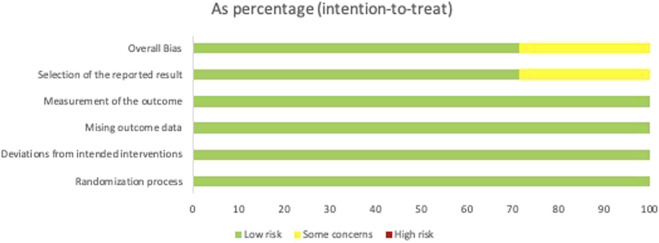
Summary plot of the risk-of-bias assessment of RCTs using RoB 2.0. RoB 2.0, Risk of Bias 2.0; RCTs, randomized controlled trials.

Using the RoB 2.0 tool, the risk-of-bias assessment is summarized in Figures 2, 3. Among all seven included studies, the randomization process (100%), deviation from intended interventions (100%), missing outcome data (100%), and measurement of the outcome (100%) were judged to be at low risk of bias. However, some concerns related to the risk of bias in the selection of the reported result were identified in Sadda et al. (2023) and Kang et al. (2024).

### 3.3 Meta-analysis of outcomes

#### 3.3.1 Primary outcomes

##### 3.3.1.1 Change in BCVA from baseline to endpoint

**FIGURE 4:**
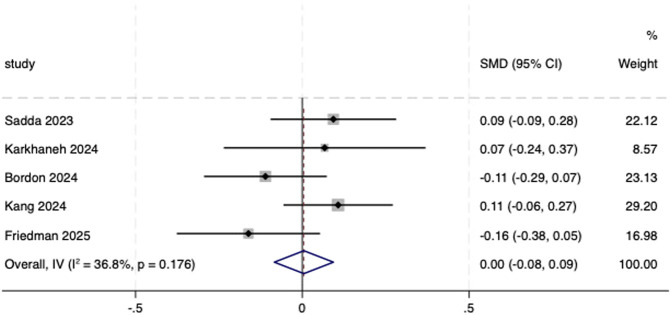
Forest plot of BCVA. BCVA, best-corrected visual acuity.

Five included studies (Sadda et al., 2023; Karkhaneh et al., 2024; Bordon et al., 2024; Kang et al., 2024; Friedman et al., 2025) reported the change in BCVA from baseline to endpoint and were therefore included in the meta-analysis. A fixed-effects model was utilized (*I* ^*2*^ = 36.8%, *P* = 0.176). The result showed no statistical difference between aflibercept biosimilars and the reference aflibercept (SMD = 0.00, 95% CI: −0.08, 0.09; Figure 4).

##### 3.3.1.2 Serious ocular and non-ocular adverse events

**FIGURE 5:**
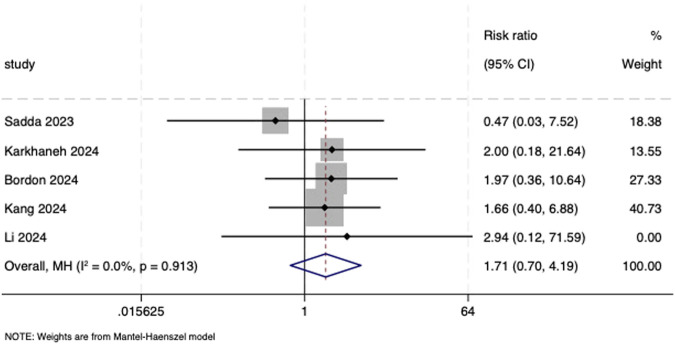
Forest plot of serious ocular adverse events.

**FIGURE 6:**
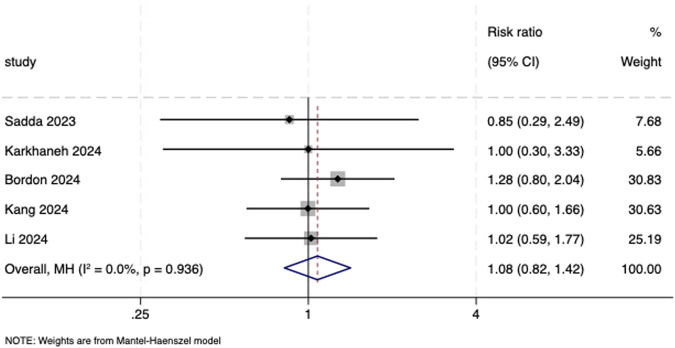
Forest plot of serious non-ocular adverse events.

Five included studies (Sadda et al., 2023; Karkhaneh et al., 2024; Bordon et al., 2024; Kang et al., 2024; Li et al., 2024) reported the number of serious ocular and non-ocular adverse events and were therefore included in the meta-analysis. Results from the fixed-effects model showed almost no difference in the incidence of serious ocular and non-ocular adverse events between the aflibercept biosimilars and the reference aflibercept (RR = 1.71, 95% CI: 0.70, 4.19; *I* ^*2*^ = 0.0%, *P* = 0.913; RR = 1.08, 95% CI: 0.82, 1.42; *I* ^*2*^ = 0.0%, *P* = 0.936, respectively; Figures 5, 6). It is worth noting that the major serious ocular adverse event reported was retinal hemorrhage. Major serious non-ocular adverse events included cardiac disorders; nervous system disorders; injury, poisoning, and procedural complications; gastrointestinal disorders; and respiratory, thoracic, and mediastinal disorders.

##### 3.3.1.3 TEAEs

**FIGURE 7:**
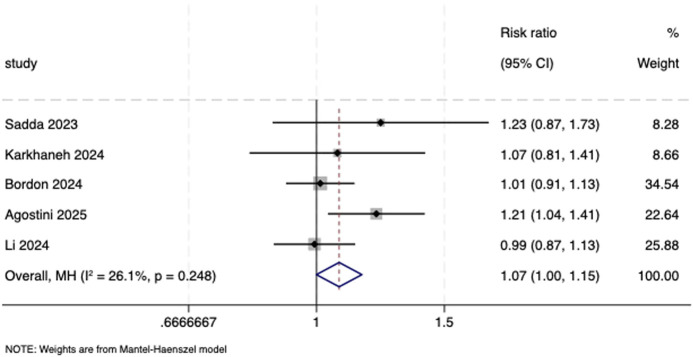
Forest plot of TEAEs. TEAEs, treatment-emergent adverse events.

Five studies (Sadda et al., 2023; Karkhaneh et al., 2024; Bordon et al., 2024; Agostini et al., 2025; Li et al., 2024) were incorporated into the meta-analysis of TEAEs. Results from the fixed-effects model indicated directionally higher but not statistically significant results (RR = 1.07, 95% CI: 1.00, 1.15; *I* ^*2*^ = 26.1%, *P* = 0.248; Figure 7).

#### 3.3.2 Secondary outcomes

##### 3.3.2.1 Reduction in CST from baseline to endpoint

**FIGURE 8:**
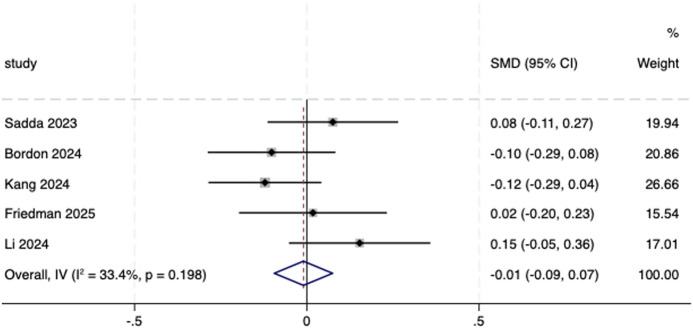
Forest plot of CST. CST, central subfield thickness.

Five included studies (Sadda et al., 2023; Bordon et al., 2024; Kang et al., 2024; Friedman et al., 2025; Li et al., 2024) reported the reduction in CST from baseline to endpoint and were therefore included in the meta-analysis. A fixed-effects model was utilized (*I* ^*2*^ = 33.4%, *P* = 0.198). The result showed no statistical difference between aflibercept biosimilars and the reference aflibercept (SMD = −0.01, 95% CI: −0.09, 0.07; Figure 8).

##### 3.3.2.2 Decrease in CNV from baseline to endpoint

**FIGURE 9:**
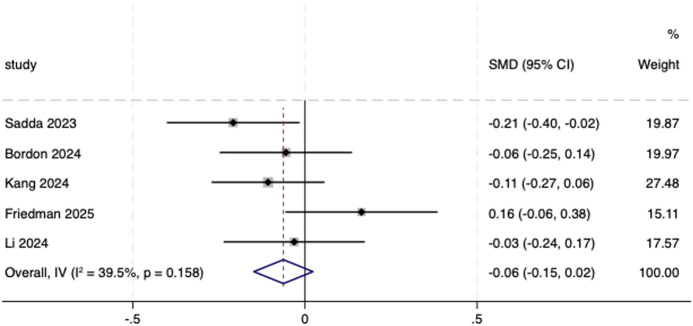
Forest plot of CNV. CNV, choroidal neovascularization.

Five included studies (Sadda et al., 2023; Bordon et al., 2024; Kang et al., 2024; Friedman et al., 2025; Li et al., 2024) reported a decrease in CNV lesion area and leakage activity from baseline to endpoint, primarily assessed through optical coherence tomography (OCT) and fluorescein angiography (FA). Both the biosimilar and reference aflibercept groups demonstrated comparable decreases in lesion size and leakage extent (SMD = −0.06, 95% CI: −0.15 to 0.02; Figure 9). No statistically significant difference was observed between the groups.

##### 3.3.2.3 Cumulative incidence of ADA

**FIGURE 10:**
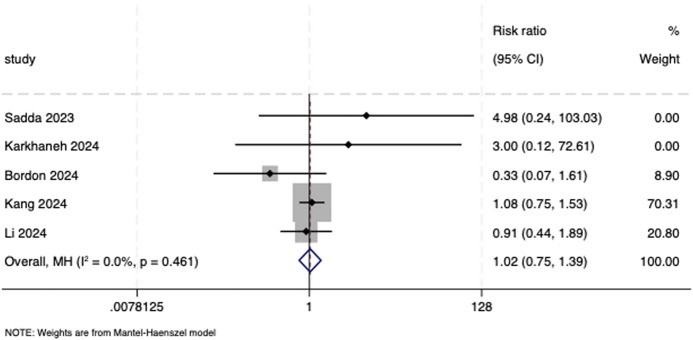
Forest plot of cumulative incidence of ADA. ADA, anti-drug antibodies.

Five trials (Sadda et al., 2023; Karkhaneh et al., 2024; Bordon et al., 2024; Kang et al., 2024; Li et al., 2024) reported the cumulative incidence of ADA and were therefore included in the meta-analysis. A fixed-effects model was utilized (*I* ^*2*^ = 0.0%, *P* = 0.461). The result showed little to no difference in the risk of ADA between the biosimilar aflibercept and the reference product (RR = 1.02, 95% CI: 0.75, 1.39; Figure 10).

#### 3.3.3 Sensitivity analysis and publication bias

Sensitivity analysis was conducted on seven studies, which showed reliable and stable results. These results are shown in Supplementary Figures S1–S14. We used the funnel plot to detect publication bias for seven studies, and no sign of asymmetry was found. In addition, Egger’s test did not suggest the existence of publication bias, as shown in Supplementary Figures S1–S14.

## 4 GRADE assessment

Although the included randomized controlled trials are considered to provide the highest level of evidence, the quality of evidence for the outcomes should be interpreted with caution. The evidence for outcomes such as BCVA, CST, and CNV was rated as low by being downgraded to the risk of bias and imprecision. The outcomes of serious ocular and non-ocular adverse events and TEAEs were defined as low and very low evidence because the uncertainty is even greater regarding the long-term risks of rare and serious adverse events and the confidence interval for the effect size is wide (Table 3).

**TABLE 3:** GRADE analysis and certainty of evidence.

Certainty assessment							No. of patients		Effect		Certainty	Importance
No. of studies	Study design	Risk of bias	Inconsistency	Indirectness	Imprecision	Other considerations	Aflibercept biosimilar	Reference aflibercept	Relative (95% CI)	Absolute (95% CI)		
Change in BCVA from baseline to endpoint												
5	Randomized trials	Serious^a^	Not serious	Not serious	Serious^b^	None	1,086	937	-	SMD 0 SD (0.08 lower to 0.09 higher)	⊕⊕⃝⃝, low^a^ ^,^ ^b^	Critical
Serious ocular adverse events												
5	Randomized trials	Serious^a^	Not serious	Not serious^c^	Serious^b^ ^,^ ^d^	None	13/1,019 (1.3%)	7/655 (1.1%)	RR 1.71 (0.70–4.19)	1 more per 100 (from 0 fewer to 3 more)	⊕⊕⃝⃝, low^a^ ^,^ ^b^ ^,^ ^c^ ^,^ ^d^	Critical
Serious non-ocular adverse events												
5	Randomized trials	Serious^a^	Not serious	serious^c^	Serious^b^ ^,^ ^d^	None	99/1,019 (9.7%)	86/655 (13.1%)	RR 1.08 (0.82–1.42)	11 more per 1,000 (from 24 fewer to 55 more)	⊕⃝⃝⃝, very low^a^ ^,^ ^b^ ^,^ ^c^ ^,^ ^d^	Critical
TEAEs												
5	Randomized trials	Serious^a^	Not serious	Not serious^c^	Serious^b^ ^,^ ^d^	None	577/937 (61.6%)	467/814 (57.4%)	RR 1.07 (1.00–1.15)	40 more per 1,000 (from 0 fewer to 86 more)	⊕⊕⃝⃝, low^a^ ^,^ ^b^ ^,^ ^c^ ^,^ ^d^	Critical
Change in CST from baseline to endpoint												
6	Randomized trials	Serious^a^	Not serious	Not serious	Serious^b^	None	1,246	1,112	-	SMD 0.01 SD lower (0.09 lower to 0.07 higher)	⊕⊕⃝⃝, low^a^ ^,^ ^b^	Critical
Change in CNV from baseline to endpoint												
5	Randomized trials	Serious^a^	Not serious	Not serious	Serious^b^	None	1,119	1,005	-	SMD 0.06 SD lower (0.15 lower to 0.02 higher)	⊕⊕⃝⃝, low^a^ ^,^ ^b^	Critical

Abbreviations: BCVA, best-corrected visual acuity; TEAEs, treatment-emergent adverse events; CNV, choroidal neovascularization; CST, central subfield thickness; CI, confidence interval; RR, risk ratio; SMD, standardized mean difference.

^a^ Sadda et al. (2023) and Kang et al. (2024) were assessed as having some concerns.

^b^ Imprecision due to wide confidence intervals for key outcome.

^c^ The uncertainty is even greater regarding the long-term risks of rare and serious adverse events.

^d^ The confidence interval for the effect size is wide.

## 5 Discussion

This systematic review and meta-analysis focuses on the safety and efficacy of aflibercept biosimilars compared with the reference aflibercept in the treatment of patients with nAMD. In this study involving seven RCTs with 2,829 participants, the aflibercept biosimilars demonstrated comparable efficacy to the reference aflibercept. The results showed no significant differences in BCVA, CST, and CNV lesions between the two groups at study endpoints in terms of visual improvement and anatomical outcomes. In addition to BCVA and CST, the regression of CNV lesion area and its associated leakage represents another important anatomical marker of treatment response in nAMD. Although the pooled analysis did not demonstrate statistically significant differences between groups, both aflibercept biosimilars and the reference product were associated with reductions in CNV lesion size and decreased fluorescein leakage. These changes, observed across multiple trials including SB15, SDZ-AFL, and AVT06, were assessed using FA and OCT and reflect the suppression of pathological angiogenesis and vascular permeability. These consistent anatomical improvements further support the clinical equivalence of biosimilars and originator aflibercept in terms of anti-VEGF activity. Regarding the serious ocular and non-ocular adverse reactions, their incidence was similar in both treatment groups. Regarding the TEAEs, no statistically significant difference was found between the aflibercept biosimilars and reference aflibercept.

Minimal clinically important difference (MCID) is defined as the smallest change in the visual acuity score over 1 year that would be clinically meaningful. A study (Potter et al., 2008) has shown that in nAMD, they chose to define the MCID at a difference in mean change between the groups of 7.5 letters (equivalent to 1.5 lines) of Early Treatment Diabetic Retinopathy Study (ETDRS) acuity. Compared with the conclusion drawn in our study, in terms of BCVA, the effect size between the aflibercept biosimilars and reference aflibercept is 0.00, which is less than 7.5 letters. Therefore, there is no clinically important difference between the aflibercept biosimilars and reference aflibercept.

Beyond pooled analysis, it is noteworthy that individual biosimilar trials reported varying rates of immunogenicity and TEAEs. In the AVT06 study, the ADA-positive rate at week 52 was 66.8% in the AVT06 group and 80.5% in the reference aflibercept group, with corresponding NAb-positive rates among ADA-positive participants being 79.6% and 87.3%, respectively (Agostini et al., 2025). In contrast, the SDZ-AFL study reported an ADA incidence of only 0.9% (2/234) in the biosimilar group and 2.6% (6/231) in the reference group (Bordon et al., 2024). This discrepancy may be attributed to differences in assay sensitivity, sample collection time points, and patient populations. Regarding TEAEs, although the pooled analysis showed a slightly higher incidence with biosimilars, individual trial results varied. For instance, some biosimilars showed numerically higher TEAE rates than reference aflibercept, whereas others demonstrated comparable or even lower rates. These differences underscore the importance of product-specific evaluation and long-term pharmacovigilance to ensure consistent safety and immunogenicity profiles across biosimilars.

nAMD is a severe ocular disease that can lead to partial or complete blindness. It affects millions of people worldwide, significantly impairing quality of life and global annual economic productivity (Marques et al., 2021). The pathogenesis involves the growth of neovascularization beneath the macula; these new blood vessels leak fluid, damaging the macula and consequently causing vision loss. The first-line treatment for nAMD is anti-VEGF agents (Bressler et al., 2011; Mitchell et al., 2018), which inhibit the growth of abnormal subretinal neovascularization. Patients with nAMD require regular intravitreal injections of anti-VEGF agents. However, anti-VEGF agents are expensive, typically requiring monthly injections and regular outpatient follow-up visits to monitor treatment efficacy and determine the need for further injections, thus imposing a financial burden on both patients and healthcare systems globally (Almony et al., 2021).

However, this study has several limitations. First, the included studies involved a wide variety of aflibercept biosimilar products. Only one published randomized controlled clinical trial was identified for each type of aflibercept biosimilar, and variations in ethnicity, countries, and regions may affect the generalizability of the results. Additionally, this limitation prevents a thorough assessment of reproducibility. Second, the randomized controlled trials included in this review had follow-up durations of 52 and 56 weeks, which are insufficient to detect long-term safety or immunogenicity differences. Finally, no real-world studies were included in the analysis, which also leads to uncertainty in the results. Future studies will conduct real-world research to validate the findings of this study.

## 6 Conclusion

This meta-analysis revealed no statistically significant differences between aflibercept biosimilars and the reference aflibercept in measures including BCVA, adverse events, CST, and CNV. Thus, this study provides preliminary evidence regarding the efficacy and safety of aflibercept biosimilars relative to the reference aflibercept therapy. Future research requires more rigorous studies.
