# Supplementary figures and images for: Efficacy and safety of aflibercept biosimilars compared to aflibercept in the treatment of neovascular age-related macular degeneration: a systematic review and meta-analysis
Source: Front Pharmacol. 2026 Feb 17;17:1719493. doi: 10.3389/fphar.2026.1719493 (PMC12953524; doi:10.3389/fphar.2026.1719493)

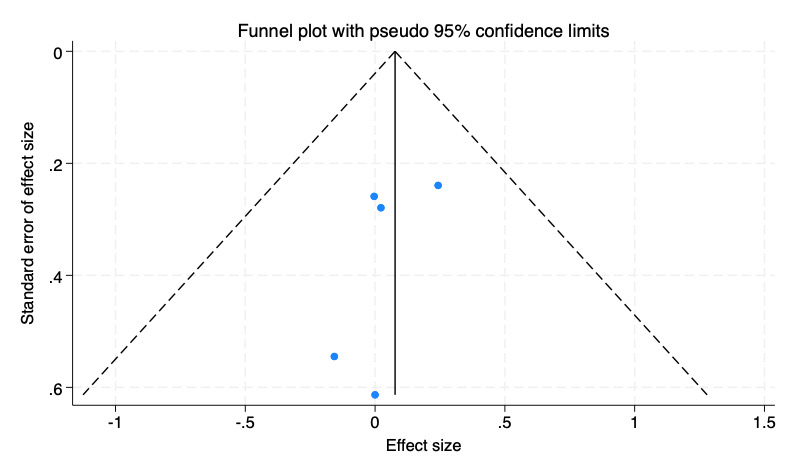

Supplement: Supplementary file 1 [file Image3.jpeg]

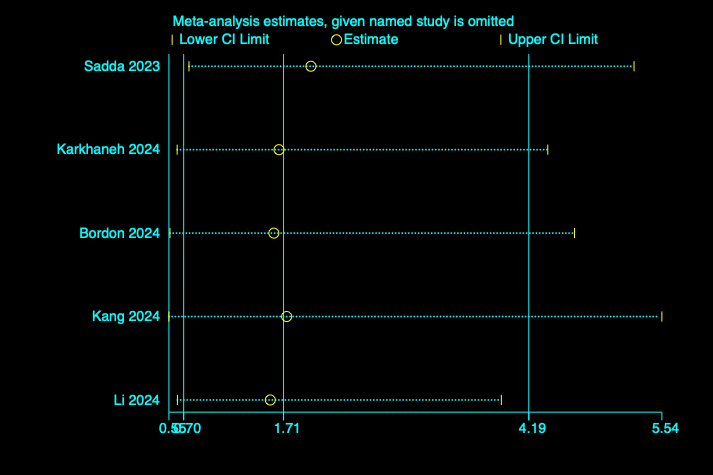

Supplement: Supplementary file 2 [file Image9.jpeg]

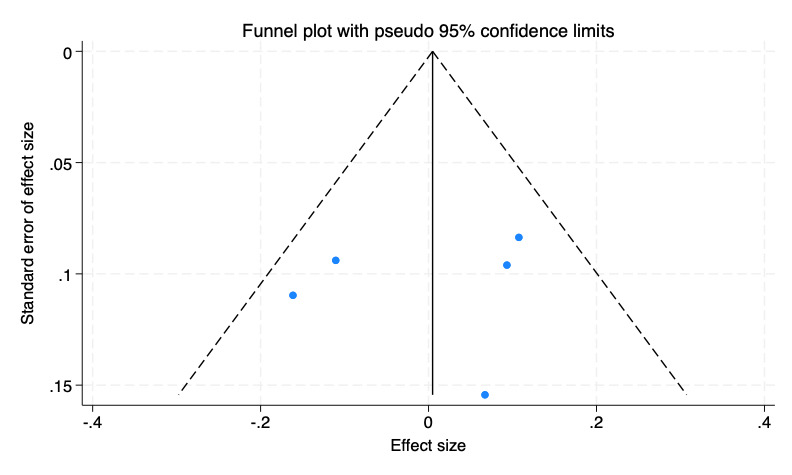

Supplement: Supplementary file 3 [file Image1.jpeg]

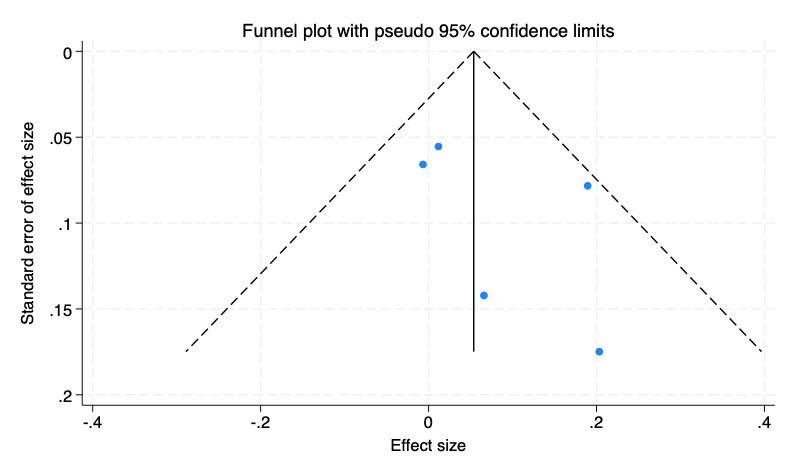

Supplement: Supplementary file 4 [file Image4.jpeg]

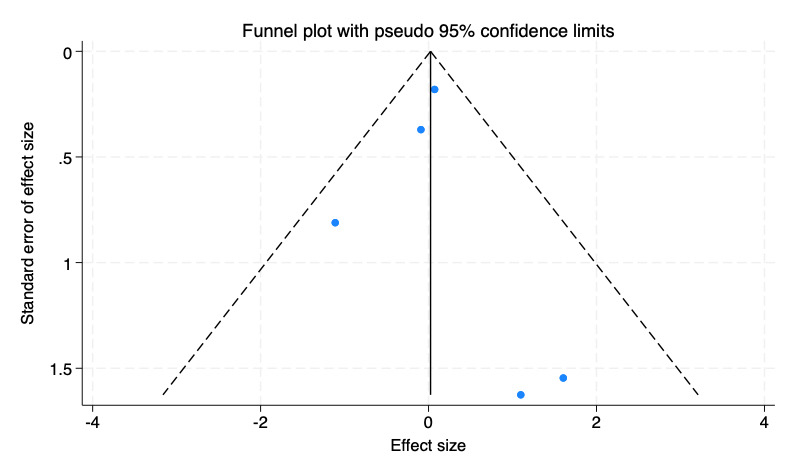

Supplement: Supplementary file 5 [file Image7.jpeg]

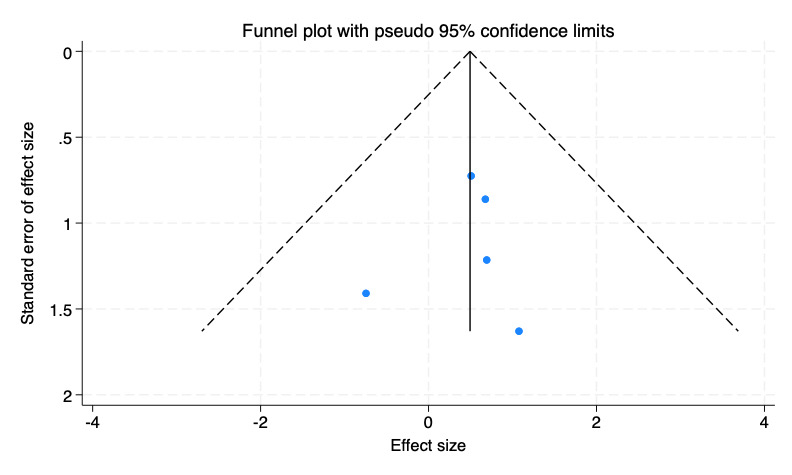

Supplement: Supplementary file 6 [file Image2.jpeg]

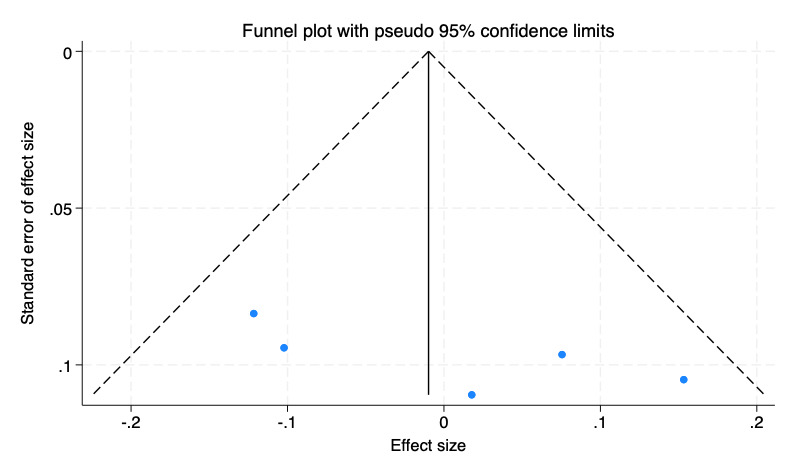

Supplement: Supplementary file 7 [file Image5.jpeg]

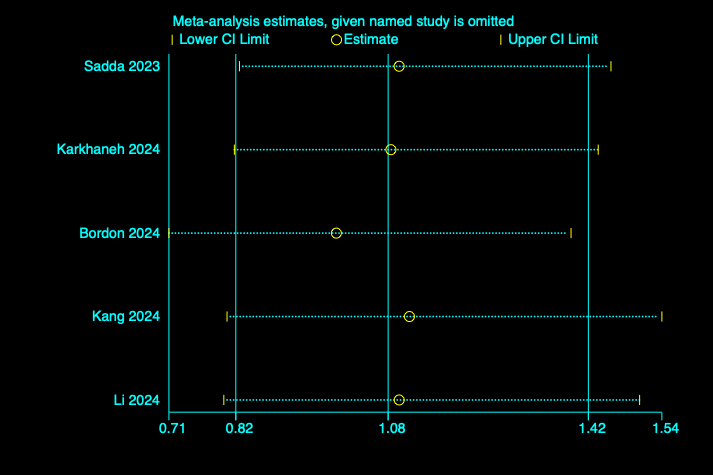

Supplement: Supplementary file 8 [file Image10.jpeg]

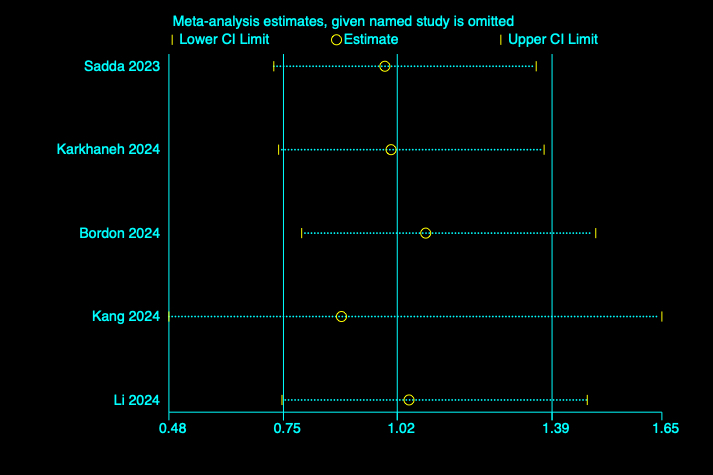

Supplement: Supplementary file 9 [file Image14.jpeg]

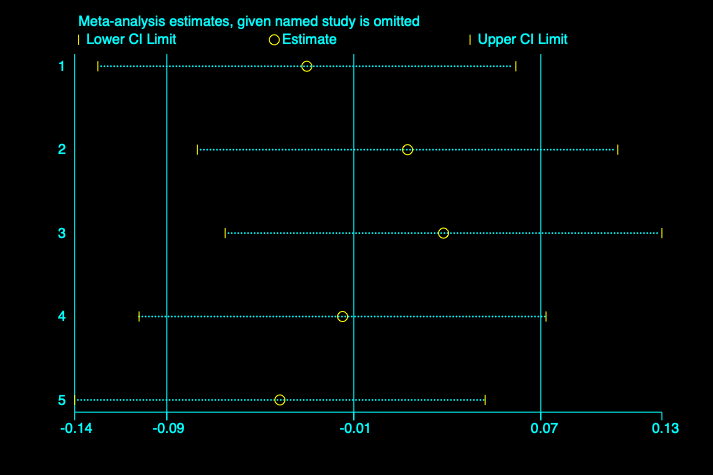

Supplement: Supplementary file 10 [file Image12.jpeg]

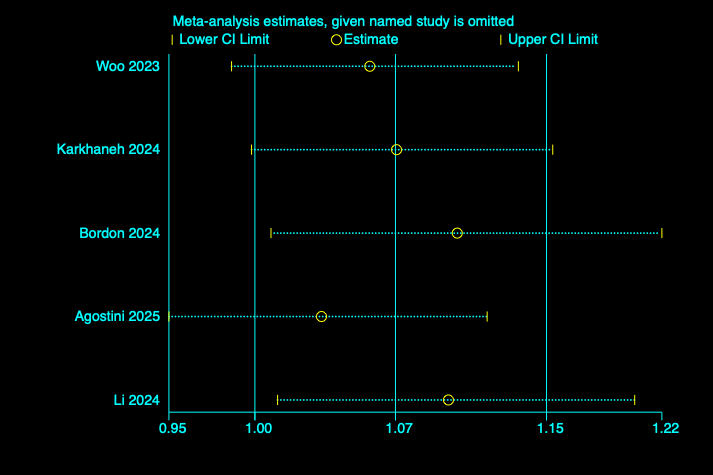

Supplement: Supplementary file 11 [file Image11.jpeg]

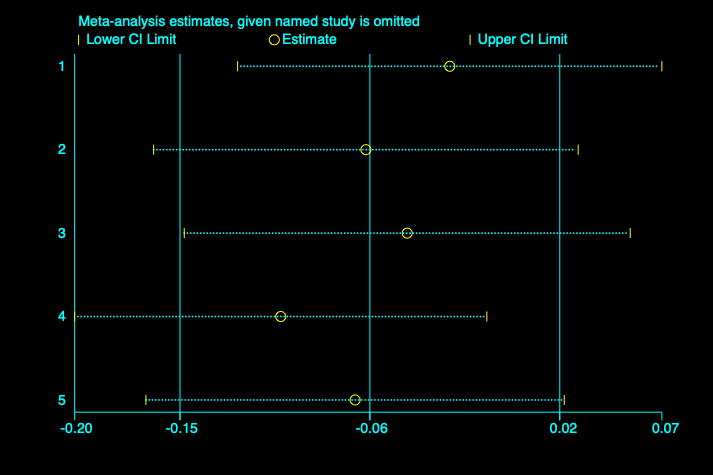

Supplement: Supplementary file 12 [file Image13.jpeg]

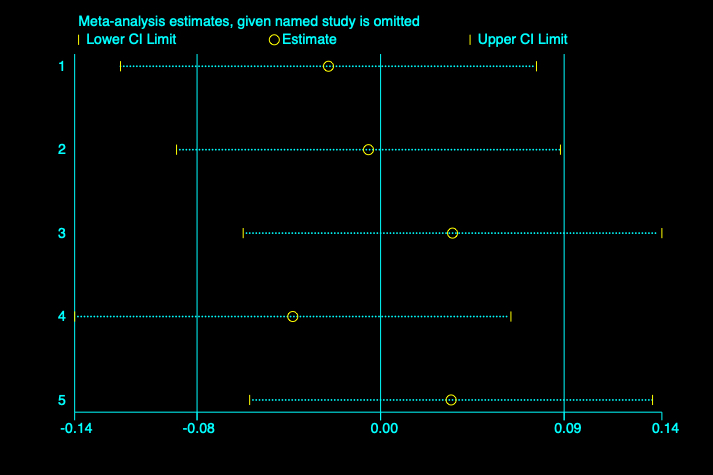

Supplement: Supplementary file 13 [file Image8.jpeg]

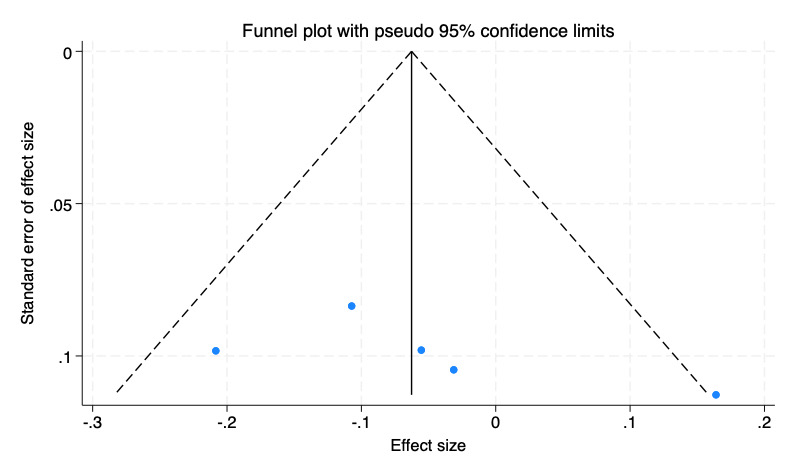

Supplement: Supplementary file 14 [file Image6.jpeg]
